# Bats Use Magnetite to Detect the Earth's Magnetic Field

**DOI:** 10.1371/journal.pone.0001676

**Published:** 2008-02-27

**Authors:** Richard A. Holland, Joseph L. Kirschvink, Thomas G. Doak, Martin Wikelski

**Affiliations:** 1 Department of Ecology and Evolutionary Biology, Princeton University, Princeton, New Jersey, United States of America; 2 Institute of Integrative and Comparative Biology, University of Leeds, Leeds, United Kingdom; 3 Division of Geological and Planetary Sciences, California Institute of Technology, Pasadena, California, United States of America; Georgia State University, United States of America

## Abstract

While the role of magnetic cues for compass orientation has been confirmed in numerous animals, the mechanism of detection is still debated. Two hypotheses have been proposed, one based on a light dependent mechanism, apparently used by birds and another based on a “compass organelle” containing the iron oxide particles magnetite (Fe_3_O_4_). Bats have recently been shown to use magnetic cues for compass orientation but the method by which they detect the Earth's magnetic field remains unknown. Here we use the classic “Kalmijn-Blakemore” pulse re-magnetization experiment, whereby the polarity of cellular magnetite is reversed. The results demonstrate that the big brown bat *Eptesicus fuscus* uses single domain magnetite to detect the Earths magnetic field and the response indicates a polarity based receptor. Polarity detection is a prerequisite for the use of magnetite as a compass and suggests that big brown bats use magnetite to detect the magnetic field as a compass. Our results indicate the possibility that sensory cells in bats contain freely rotating magnetite particles, which appears not to be the case in birds. It is crucial that the ultrastructure of the magnetite containing magnetoreceptors is described for our understanding of magnetoreception in animals.

## Introduction

The role of the earth's magnetic field for orientation and navigation has been confirmed in several animal taxa, including birds [1], [2], insects [3], lobsters [4], salamanders [5], turtles [6], fish [7] and mammals , including–most recently–bats [8], [9]. However, the mechanisms by which animals detect the Earth's magnetic field have remained controversial. Two independent hypotheses have been proposed: a light dependent mechanism [10] and one based on the biogenic ferromagnetic mineral magnetite and/or its solid-solution oxidized equivalent maghemite [11]. Whilst these two mechanisms have in the past been argued to be in competition [12], current behavioral evidence suggests that some animals might use both mechanisms [13].

In some birds and amphibians, light may affect information used for compass orientation [5], [14]. In other taxa the compass mechanism generally appears to be light-independent: magnetotactic bacteria and some protists passively align to the magnetic field due to intracellular magnetite [15], [16]; turtles and lobsters can orient in complete darkness [17], and salmon use magnetite to detect changes in magnetic intensity as well as for compass use [18]. Mole rats (*Fukomys anselli*), the only mammal group studied so far; orient by a polarity based compass which suggests the use of magnetite, [19] and recent experiment involving anaesthesia of the eye links magnetite to the detection process [20].

Recently, the big brown bat, *Eptesicus fuscus,* was shown to possess a magnetic compass for homing [8], but the mechanistic nature of this compass remained unknown. From an ecological perspective bats compare closely with birds in terms of their ability to make rapid, wide ranging movements. Also like birds, some bats make seasonal migrations spanning many 1000's of km [21]. Previous experiments have indicated that vision is essential for homing in bats beyond the range of their echolocation system, suggesting the possibility that bats have a light mediated magnetic compass [22]. However, bats have also been shown to have magnetite in their bodies [23], although it has not yet been linked to sensory neurons as in other vertebrates [18].

Since the discovery that magnetotactic bacteria biologically precipitate magnetite of single-domain size [15], [24], [25] it has been proposed that this could form the model for a “compass organelle” [11]. Single-domain magnetite is uniformly and spontaneously magnetized at its maximum value, and has a stable magnetic moment strong enough to align spontaneously in the geomagnetic field despite thermal agitation. These features make it an ideal transducer for detection of the geomagnetic field [26]. Furthermore, the magnetisation of individual crystals of magnetite must lie parallel to the easy axis of magnetization, which is typically parallel to the long axis of the crystals, and hence can be in one of two stable orientations. As these are aligned in parallel chains within the magnetotactic bacteria they can be permanently flipped between these orientations by applying a brief (<0.1 mS) strong (∼0.1 T) magnetic pulse antiparallel to the chain direction; freely-moving chains can be held in a fixed orientation relative to such a pulse by application of a constant biasing field of only a few mT. The ability of this ‘Kalmijn-Blakemore’ re-magnetisation experiment [27] to permanently convert North-seeking bacteria into South-seeking forms (and vice-versa) is a unique and definitive proof of the ferromagnetic basis of their magnetotactic response [28]. It has been proposed by Kirschvink and Gould [11] that freely moving magnetite particles could exist in eukaryote cells if a suitably sized single domain magnetite grain is held in a membrane but is free to align to the earths magnetic field. If this were linked to mechanically activated ion channels it could signal the direction of the magnetic field when certain orientations were presented (see [11] for diagrammatic representation of this proposed receptor). A strong biasing field (several to many times stronger than the local geomagnetic field) will torque the magnetosome chains into reasonable alignment with the field direction like compass needles. A short magnetic pulse of any intensity applied parallel to the biasing field should then have no effect [29]. In contrast, applying a pulse strong enough to flip the magnetization direction of the crystals, but antiparallel to the biasing field will reverse the polarity of the chain. Since polarity information is needed to determine the direction of the magnetic field, the ‘Kalmijn-Blakemore’ experiment provides a behavioral assay for an organism's use of single-domain magnetite as a method of magnetic **compass** detection for receptor cells containing freely-moving magnetosomes [28]. If the magnetite is not free to rotate then both parallel and antiparallel pulses should affect the magnetite chains.

Pulse re-magnetisation has been used to test for the presence of magnetite-based magnetoreception in birds, but the results, while confirming the role of magnetite, have been difficult to interpret due to the design of most of these experiments, in which the pulse was applied perpendicular to the only biasing field present, that of the earth [1], [30] [31]. Effects were seen only in adults, not in juvenile migratory birds, suggesting that the effect was on an experience-based system such as a geomagnetic ‘map’ rather than on the compass. In biophysical terms, the effect of a pulse applied perpendicular to the biasing field, or with no bias is less predictable, but it most likely results in magnetosome chains with a mixture of magnetic directions. This would lead to ‘kinks’ that effectively lower net magnetization, and would most likely affect organelles specialized to extract intensity information from the magnetic field used in a magnetic map [32]. Wiltschko et al. [33] report the only experiment so far on birds in which a biasing field was applied either parallel or antiparallel to a pulse, and this led to a peculiar east-west axial orientation in both groups. Their results could not be clearly attributed to an effect either on the magnetic compass or map. A pulse experiment on mole rats [34] did apply a pulse on a north-south axis but the experimental description did not make it clear whether this was parallel or antiparallel to the Earth's magnetic field (there was no biasing field present). Consequently, no definitive conclusions about whether the pulse affected polarity could be made, although behavioural responses to other tests in the same paper make compass orientation the most likely interpretation in this animal. Pulse remagnetization has also indicated the presence of a magnetite based system in bees [35] and turtles [36], but again the responses made it difficult to distinguish between a map and a compass.

Thus, the ‘Kalmijn-Blakemore’ experiment as applied to bacteria has never before been used successfully to test the presence of freely rotating magnetite particles for compass use in animals. The goal of this study was to apply this technique of pulses parallel and antiparallel to a north-south biasing field to test whether bats use freely rotating magnetite as a polarity-sensitive compass to detect the Earth's magnetic field for homing after displacement.

## Results

**Figure 1:**
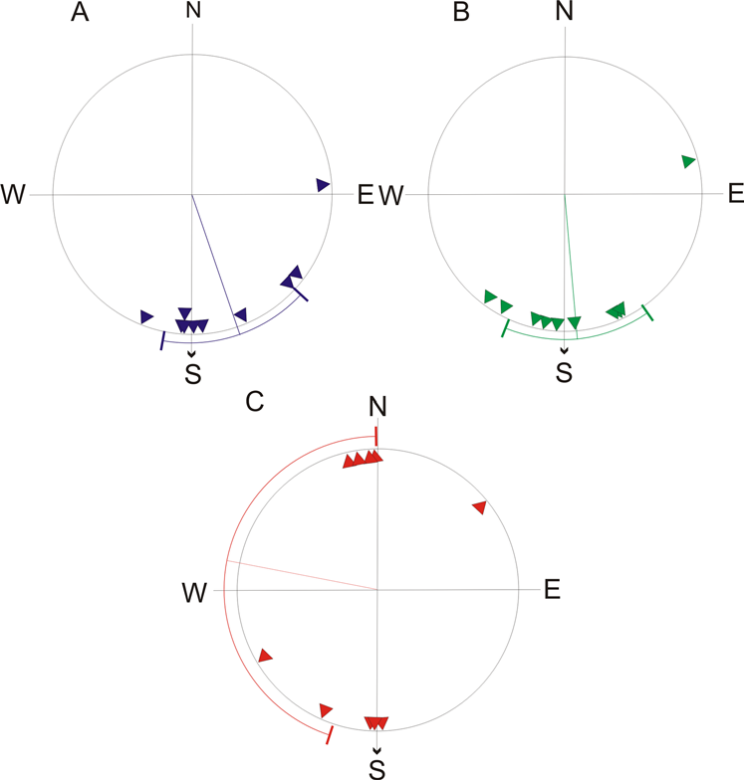
Vanishing bearings of A, control, B, parallel-pulsed and C, antiparallel pulsed bats. The mean direction and 95% confidence interval are shown. The arrow on the edge of the circle indicates the home direction.

**Figure 2:**
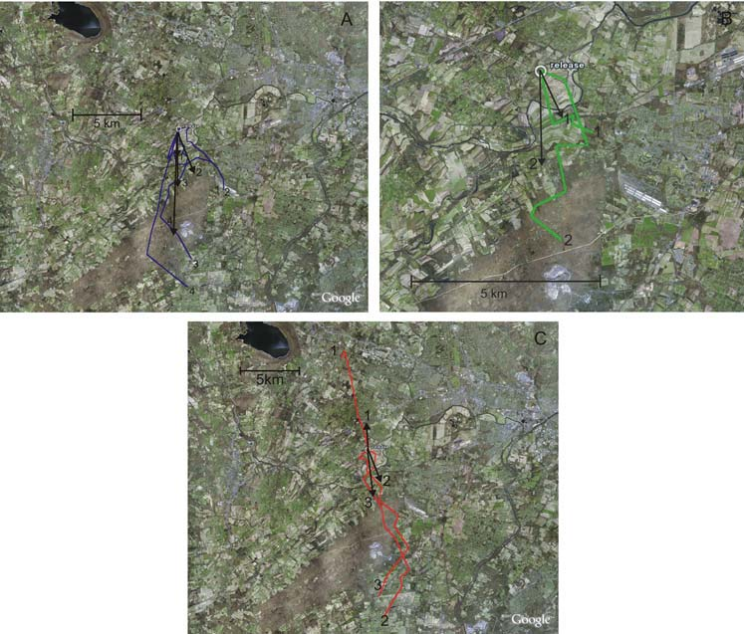
Tracks of A, control bats B, bats pulsed parallel to the biasing field and C, bats pulsed antiparallel to the biasing field. Vector arrows represent the vanishing bearing and distance. Numbers identify the track and the vanishing bearing associated with it.

Control bats (Figure 1A) were significantly oriented (Rayleigh test, Z = 7.462, p<0.0001) and the mean vector did not differ significantly from the home direction of 180° (confidence interval test, p>0.05). Bats in the parallel-pulsed group (figure 1B) were also significantly oriented (Rayleigh test, Z = 6.791, p<0.0001) and again the mean vector did not differ significantly from the home direction (confidence interval test, p>0.05). The antiparallel-pulsed group (figure 1C) were not significantly oriented (Rayleigh test, Z = 0.098, p>0.05). Table 1 shows the results in numerical form. There was a significant difference in the dispersion of the groups (dispersion test: X^2^ = 14.74, p<0.001). *Post hoc* analysis indicated a significant difference between the antiparallel-pulsed group and all other groups, but no difference between the parallel-pulsed and control groups (Dunn's test: antiparallel-pulsed vs. parallel-pulsed, p<0.05, antiparallel-pulsed vs. control: p<0.001, parallel-pulsed vs. control: p>0.05), with the antiparallel-pulsed group showing significantly more dispersion than the other groups. The antiparallel-pulsed group appears to show bimodal orientation on the north south axis, and this is supported by the significant orientation of this group if treated as axial (Rayleigh test, Z = 4.912, p<0.005). There was no significant difference between groups in the time taken to vanish from the release site (ANOVA, F = 0.524, p>0.05), nor was there a difference between the vanishing direction and the aircraft track bearing at the point of vanishing (dispersion test: U = 56, p>0.05, figures 2A, B, C). There was no significant difference in homing performance between groups (Chi Squared test, X^2^ = 2.61, p>0.05). There was also no difference between sexes in vanishing bearings (Mardia-Watson-Wheeler test: W = 0.35, p = 0.839).

**Table 1:** Orientation statistics^a^.

Pulse condition	N (ratio male/female)	Mean vector±95% confidence interval (°)	Difference
Control	7/3	163.519±22.475**	Antiparallel***
Parallel	5/5	176.999±25.786**	Antiparallel*
Anti-parallel	6/4	278.79±72.210	Parallel*
			Control***

a Significance is indicated by *.

* , p<0.05, **, p<0.01, ***, p<0.001.

Significant orientation of mean vector is by Rayleigh test. Significant difference by Kruskal-Wallace test with Dunn's test post hoc comparisons.

## Discussion

These results uniquely identify a single domain magnetite based, polarity-sensitive receptor as providing an essential component for detecting the magnetic field in the big brown bat, as the potential non-specific effects of the brief pulse (mainly from electrical induction) are the same in both the parallel and antiparallel groups. A non-specific effect of the pulse should also have produced a difference between the parallel-pulsed group and the controls, yet they were indistinguishable. Hence, the orientation of single-domain ferromagnetic particles in the bat prior to exposure to a pulse is the only viable explanation for our results. As only the antiparallel group is affected by the pulse, this indicates that the pulse changed the polarity of the magnetite. A magnetite based receptor that is responsive to polarity is a precondition for magnetic compass use by magnetite. A pulse applied in this way should not effect the detection of the magnitude of either the intensity or the inclination of the magnetic field, which are elements of the proposed geomagnetic map in birds [31], [37], [38]. We note however, that at present the mechanism by which intensity is measured by magnetite is poorly understood so at this stage an additional effect on intensity detection cannot totally discounted. Nevertheless, The results are consistent with the recent discovery that roosting bats respond to altered polarity of the magnetic field when choosing a roosting location [9]. A light-dependent compass is unable to detect the polarity of the magnetic field, as the hypothesized biophysical transduction mechanism is invariant to field polarity [13].

Not all bats exposed to an antiparallel pulse appeared to be affected, however. Indeed the behaviour of the group appears to be bimodally distributed between homeward and opposite headings and this is supported by significant orientation in the antiparallel group if it is treated as axial. If the magnetite was not free to rotate then the pulse may have caused this axial response. However as the bats used in this experiment were normally free-foraging, it is possible that some of them were familiar with the release site and simply flew home, ignoring conflicting compass information. This is consistent with previous results [8] showing that although big-brown bats are initially deflected in their homing direction by a magnetic treatment in a Helmholtz coil, many of them nevertheless found home during the release night. Alternatively we hypothesize that bats have, and use, additional directional information that they could switch to when they realised that the magnetic compass was faulty (for example an alternative compass mechanism), or that perhaps some bats weighed higher than others in a hierarchy. Previous experiments on birds have indicated that reliance on different compass mechanisms may change with age and experience [39]. It is also consistent with the results of cue conflict experiments in other animals in which different animals may use different strategies from the same release site [40]. It is unlikely that relative experience *per se* was responsible for the effect in the antiparallel group as this would have required the non random assortment of inexperienced individuals into this experimental group only. There was also no effect of sex on vanishing bearings and so the effect could not be explained in terms of a differential response between male and female bats. If the axial response in the antiparallel group is indeed due to an alternative compass mechanism or navigation strategy and not a response to the pulse *per se* then our results indicate that the magnetite in the magnetoreceptor cells is free to rotate, which would make an important distinction from birds [33] where the results do not support freely rotating magnetite.

Our data indicate that, at a fundamental physiological level, the magnetoreceptor cells that provide compass information to the animals are sensitive to magnetic polarity, and hence we can rule out cellular ultrastructures in which the ferromagnetic materials are arranged to be polarity insensitive (as suggested in ref. [11], and possibly employed in birds). A particularly simple arrangement that is compatible with our data is the coupling of one end of a magnetosome chain to mechanically-activated ion channels in a suitable receptor cell [12], [41]. Presumably these chains would be arranged to allow maximum response during normal flight in the locally steep geomagnetic field. The parallel pulse – which has no effect on the magnetic direction of the chain – would yield no change in the information returned from the system. In contrast, the antiparallel pulse would leave the chains magnetized in the opposite direction and cause them to twist away from their usual orientation, potentially providing backwards compass information consistent with the observed behavior if the magnetite is free to rotate. This makes clear predictions for future ultrastructural study of the receptor cells, if they can be found. As yet, the location of the receptor cells containing magnetite in bats is unknown and the structure of the receptor cells in any animal also remains to be determined. It is crucial to our understanding of magnetoreception by magnetite based cells that the ultrastructure of the magnetoreceptors be determined.

The role of the nervous system in magnetoreception in bats also remains to be determined. In mole rats the superior colliculus plays a role in processing of magnetic information [42]. In birds and in fish the receptor cells are located in the nasal region [18], [43] and innervated by the trigeminal nerve [44], [45], which is present in essentially all vertebrate groups.

Research on orientation and navigation in bats has lagged considerably behind other animal groups [46] but within the last 2 years a laboratory based system to test hypotheses of compass use [9] and a field based system with the possibility to test hypotheses of orientation and navigation to a known goal [8] have been developed. Although we now have good evidence for compass use by bats, the way bats locate their position with respect to their final goal (the “map” step [47]) remains to be discovered.

## Materials and Methods

### Experimental Subjects

30 Big brown bats (12 female, 18 male) *Eptesicus fuscus*, were captured at their roost in the barn at Princeton University field station from 24^th^ April to 30^th^ May 2007. When a bat was captured it was treated using the magnetic pulsing device before being displaced to the release site. At least 2 bats were used on each release night and at least 1 control was included with one of the other 2 experimental groups. No release consisted of more than 4 bats in one night. All bats were at least one year old at time of capture but age determination beyond this was not possible.

### Treatment

To administer a magnetic pulse an SCR-fired capacitive discharge unit (a SOTA™ magnetic pulser) was modified by JLK by the addition of a double-wrapped, 10 cm diameter Lee Whittling coil [48]. The coil system produced a unidirectional magnetic pulse of ∼0.1 mS duration, with peak amplitude slightly over 0.1 T, and a rise time of ∼100 nS. A pair of fine wire Helmholtz coils produced a 320 µT biasing field that could be arranged parallel or antiparallel to the pulse. The coil was set up at the field station aligned on a magnetic north-south axis and set at an angle of 67° (the inclination at this location). To receive a pulse a bat was placed in the coil with the longditudinal axis of their body, i.e the front of the head, facing magnetic north until an audible pulse was emitted and then removed. Each bat received one of 3 conditions. 1) Antiparallel pulse: the coil was aligned so that the direction of the pulse was opposite that of the biasing field (which was always oriented in the same direction as the Earth's magnetic field). In bacteria, this treatment reverses the normal swimming direction, due to the normal magnetisation of the magnetite being re-polarized in the opposite direction. 2) Parallel: the pulse was in the same direction as the biasing field. This treatment has no effect on the swimming direction of bacteria. 3) Control: the currents in the double-wrapped coil were set in opposite directions, so even though the pulse mechanism was still audible, and the same current pulse was in the circuit, no magnetic pulse was administered to the animals in the coil. The biasing field was still present in this treatment.

### Release procedure

Bats were displaced to a site at the edge of Neshanic Valley golf course, New Jersey, 20 km north of their home by car and were fitted with a 0.5 g 164 MHz radio-transmitter attached to shaved skin between the shoulders with vet bond glue, just prior to release. An activated transmitter was measured using a Handheld Sampling inc. 2-axis magnetometer (resolution 100 nT), with less than 1000 nT effect of the background Earth's magnetic field at 1 cm from the sensor and no effect on the Earth's background field 2 cm from the sensor (the approximate distance from the bat's head when attached). Previous experiments using these transmitters have demonstrated the use of a magnetic compass in both bats and birds [8], [49]. Bats were fed mealworms and water before release. Each bat was released by allowing it to fly freely from the hand and then its orientation direction was monitored using a receiver (AR8200) and handheld antenna from the release site and when the signal disappeared the direction it had vanished in was recorded, along with the time taken to vanish. This is the radio tracking equivalent of a visual vanishing bearing as used extensively in navigation research on homing pigeons [50]. 9 bats were also tracked by a small airplane (Cessna 152 or 172) until they returned to the home roost or stopped moving for more than one hour. Using a two antenna setup on the aircraft bats were located (±100 m) every several minutes and the flight path later reconstructed from the GPS waypoints recorded at these locations. A receiver at the home roost allowed us to record when a bat returned if it had to be abandoned because of lack of movement during the tracking period. Releases were performed on nights when wind speed was less than 5 km/h. Bats were released in a pseudo random order to avoid always releasing one condition first during a release night. Homing performance was measured in a presence/absence manner, recording whether the bat homed during the life of the radio transmitter.

### Statistical analysis

Vanishing bearings were analysed for directionality using the Rayleigh test, and for orientation in the home direction by the V-test [51]. Differences between groups were analysed using the variance test [51], [52], where each data point is subtracted from its sample mean to give a value between 0–180°and then this value was compared to those of the other groups using a linear Kruskal-Wallis test in the case of 3 or more independent samples or the Mann-Whitney test in the case of 2 independent samples. For the Kruskal-Wallis test, if the overall difference between groups was significant then post hoc analysis was performed using Dunn's test. Tracks were constructed from GPS waypoints in Google Earth. The vanishing bearings were compared to the direction of the track at that point in time to check that they were not significantly different, also using the variance test.

### Approval

These experiments were approved by Princeton University Institutional Animal Care and Use Committee and by the New Jersey Fish and Wildlife Services.
